# Pseudoexfoliation and Cataract Syndrome Associated with Genetic and Epidemiological Factors in a Mayan Cohort of Guatemala

**DOI:** 10.3390/ijerph18147231

**Published:** 2021-07-06

**Authors:** Patrice M. Hicks, Elizabeth Au, William Self, Benjamin Haaland, Michael Feehan, Leah A. Owen, Adam Siedlecki, Elizabeth Nuttall, Deborah Harrison, Andrew L. Reynolds, John H. Lillvis, Sandra Sieminski, Julia P. Shulman, Margarita Barnoya, Juan Jose Noguera Prera, Orlando Gonzalez, Maureen A. Murtaugh, Lloyd B. Williams, Michael H. Farkas, Alan S. Crandall, Margaret M. DeAngelis

**Affiliations:** 1 Department of Population Health Sciences, University of Utah School of Medicine, Salt Lake City, UT 84108, USA; patrice.hicks@hsc.utah.edu (P.M.H.); ben.haaland@hsc.utah.edu (B.H.); michael.Feehan@kantar.com (M.F.); leah.owen@hsc.utah.edu (L.A.O.); maureen.murtaugh@hsc.utah.edu (M.A.M.); 2 Department of Ophthalmology and Visual Sciences, University of Utah School of Medicine, Salt Lake City, UT 84132, USA; wjself@moffatfarm.com (W.S.); elizabeth.nuttall@hsc.utah.edu (E.N.); deborah.harrison@hsc.utah.edu (D.H.); alan.crandall@hsc.utah.edu (A.S.C.); 3 Department of Ophthalmology, Jacobs School of Medicine and Biomedical Engineering, SUNY—University at Buffalo, Buffalo, NY 14214, USA; elizabethdgeorge@gmail.com (E.A.); as222@buffalo.edu (A.S.); alr23@buffalo.edu (A.L.R.); jhlillvi@buffalo.edu (J.H.L.); sandrafe@buffalo.edu (S.S.); mhfarkas@buffalo.edu (M.H.F.); 4 Kantar, LLC, New York, NY 10007, USA; 5 VA Western New York Healthcare System, Buffalo, NY 14215, USA; 6 Department of Ophthalmology, New York Medical College, Valhalla, NY 10595, USA; jshulman03@gmail.com; 7 Salama Lions Eye Club Hospital, Salama 150001, Guatemala; engelbar@intelnett.com (M.B.); juanchonope@yahoo.com.mx (J.J.N.P.); orlandgpicado@gmail.com (O.G.); 8 Department of Ophthalmology, Duke University, Durham, NC 27710, USA; lloyd.williams@duke.edu

**Keywords:** genetic epidemiology, genetic, epidemiology, cataract, pseudoexfoliation syndrome, isolated population, global health, eye care

## Abstract

The Mayan population of Guatemala is understudied within eye and vision research. Studying an observational homogenous, geographically isolated population of individuals seeking eye care may identify unique clinical, demographic, environmental and genetic risk factors for blinding eye disease that can inform targeted and effective screening strategies to achieve better and improved health care distribution. This study served to: (a) identify the ocular health needs within this population; and (b) identify any possible modifiable risk factors contributing to disease pathophysiology within this population. We conducted a cross-sectional study with 126 participants. Each participant completed a comprehensive eye examination, provided a blood sample for genetic analysis, and received a structured core baseline interview for a standardized epidemiological questionnaire at the Salama Lions Club Eye Hospital in Salama, Guatemala. Interpreters were available for translation to the patients’ native dialect, to assist participants during their visit. We performed a genome-wide association study for ocular disease association on the blood samples using Illumina’s HumanOmni2.5-8 chip to examine single nucleotide polymorphism SNPs in this population. After implementing quality control measures, we performed adjusted logistic regression analysis to determine which genetic and epidemiological factors were associated with eye disease. We found that the most prevalent eye conditions were cataracts (54.8%) followed by pseudoexfoliation syndrome (PXF) (24.6%). The population with both conditions was 22.2%. In our epidemiological analysis, we found that eye disease was significantly associated with advanced age. Cataracts were significantly more common among those living in the 10 districts with the least resources. Furthermore, having cataracts was associated with a greater likelihood of PXF after adjusting for both age and sex. In our genetic analysis, the SNP most nominally significantly associated with PXF lay within the gene KSR2 (*p* < 1 × 10^−5^). Several SNPs were associated with cataracts at genome-wide significance after adjusting for covariates (*p* < 5 × 10^−8^). About seventy five percent of the 33 cataract-associated SNPs lie within 13 genes, with the majority of genes having only one significant SNP (5 × 10^−8^). Using bioinformatic tools including PhenGenI, the Ensembl genome browser and literature review, these SNPs and genes have not previously been associated with PXF or cataracts, separately or in combination. This study can aid in understanding the prevalence of eye conditions in this population to better help inform public health planning and the delivery of quality, accessible, and relevant health and preventative care within Salama, Guatemala.

## 1. Introduction

### 1.1. Pseudoexfoliation

Pseudoexfoliation syndrome (PXF) was first described in 1917 by John G. Lindberg [1,2,3]. The term pseudoexfoliation is used to distinguish from the similarly appearing, but uncommon and clinically different exfoliation syndrome of anterior capsular damage occurring in extreme infrared radiation exposure in glass blowers [1,2,3]. PXF is a systemic disease in which tissues in the body accumulate white/gray material consistent with extrafibrillar material [1,2,3]. In the eye, PXF material accumulates chronically and progressively in both extraocular and intraocular tissue due to excess production and/or decreased breakdown of elastic microfibrillar components [4]. This material is insoluble within the eye and floats within the aqueous humor and deposits on the iris, lens and zonules where it forms a pathognomonic ring pattern on the anterior lens capsule [5]. The research and clinical focus of PXF is generally within the eye [6]. PXF has also been associated with other systemic diseases such as dementia, sensory hearing loss, coronary disease, and hypertension [7,8,9]. Systemic disease co-occurs with PXF due to the abnormal breakdown and accumulation of the extrafibrillar material in both the blood vessels and the visceral organs [7,8,9].

Epidemiological risk factors for PXF include older age [10,11,12], female sex [13,14], race and ethnicity [15], environmental factors such as elevation and sun exposure [16,17], and diet, such as coffee consumption and folate deficiency [18,19,20,21]. Those who are older (40 years or over) are at a greater risk for developing PXF [10,11,12]. To date, only twelve individuals under the age of 40 have been found to have PXF [18,22,23]. The presence of PXF has been reported to be as high as 25% in people aged over 60 in some populations from Iceland. PXF is most commonly found in Icelanders, Finns, Russians, and Lapps residing in Novosibirsk, with the prevalence being 21% [24]. PXF may also contribute to 25% of primary open-angle glaucoma worldwide for all people aged 60 years and older [18]. These prevalence rates include 1.73% in a Congolese population [25], 2.8% in a Singaporean population [26], 2.3% in the Blue Mountain Eye Study [14], 38% in the Navajo American Indian population [27], 4.4% in a population in Peru [28], and no documented cases in the Greenland indigenous population [13,29]. Further studies of the individual population-level prevalence of PXF and its associations are needed to further understand PXF globally. Overall, the prevalence may be difficult to assess because PXF is often missed on examination, particularly in those following cataract surgeries. Cataract surgery is common in patients over 60 years old; therefore, PXF is much more challenging to observe and diagnose due to the removal of much of the anterior lens capsule and PXF material in the anterior chamber during surgery [4]. Women are also more likely to develop PXF compared to men [13,14]. This may be because women tend to live longer than men, and PXF development increases with age [30,31,32]. Women in low-resource countries or geographic regions may have more morbidity due to PXF because of less access to detection measures of PXF than men living in the same countries or region [33,34]. In addition to demographic risk factors, environmental risk factors have also been previously associated with PXF. These factors include living in places with a higher altitude and elevation [16,17], the amount of time spent outdoors, and solar exposure [17,35,36,37]. Dietary factors that have been linked to an increased risk of PXF include being non-vegetarian with high fish consumption [19], high coffee intake [19,20], and low folate intake [21].

Genetics are also a risk factor for PXF. Two studies have demonstrated that Icelandic monozygotic twins have a higher degree of concordance, with five out of eight twin pairs who were 55 years old having PXF [38,39]. The gene which is consistently associated with PXF is LOXL1 [40,41,42,43]. An additional gene that has commonly been associated with PXF is CACNA1A [44,45]. Studies have identified additional genes that are risk factors for PXF, including SEMA6A, RBMS3, TMEM136-ARHGEF12, AGPAT1, SOD2, ALDH1A1, and FLT1-POMP [45,46]. All the reported SNPs in the aforementioned genes increase the risk of PXF, except the protective variant: p.Tyr407Phe in LOXL1. p.Tyr407Phe in the LOXL1 variant was found to protect from the risk of PXF in 11,845 individuals from nine countries (United States, Japan, Greece, Italy, Russia, Mexico, South Africa, India and Pakistan) that included both men and women [47].

### 1.2. Cataracts

Cataracts are a clouding of the lens in the eye, which can impact vision [48]. The estimation for the number of people with cataracts in the aging population (≥60 years of age) around the world is 95 million [49]. Cataracts are the leading cause of blindness in both middle and low-resourced countries and may also develop due to disease status, such as diabetes and glaucoma [49,50,51]. Cataracts are treated by surgical lens extraction [52,53]. Although cataract surgery is very successful, in areas where there are limited resources, including access to care, there can be many barriers impacting surgical outcomes. [54,55]. For example, for those that live in low-resource areas, there can be cultural, social, and economic factors impacting cataract outcomes, including countries such as Nepal, China, Madagascar, Kenya, Ghana, the Philippines and Bangladesh [34,56,57]. Ocular symptoms of cataracts include double vision, seeing halos in the line of vision, light sensitivity, clouded/blurred vision, the need for additional light when reading and additional activities, and frequent changes in prescription for contact lenses or glasses [58]. Cataracts are diagnosed by an eye care professional and usually develop slowly; many cases do not interfere with vision initially, but over time will need to be addressed to maintain vision [59,60].

Epidemiological risk factors for cataracts include age [61], sex [34,62,63], living in a low-resourced area [56,57,61,64], educational status [65], smoking [66,67], diabetes [50,68], and environmental factors [69]. Cataracts may appear in the eye when a patient is in their 40s or 50s, although may not start to impact their vision until they are in their 60s [58]. Globally, women have a greater burden of cataracts compared to men, possibly due to their longer life spans [62]. Women live longer than men, and cataracts are associated with aging. In addition, women are less likely to receive cataract surgery as compared to men in low-resourced countries [70,71,72]. The environmental factors which are most consistently associated with cataracts are sunlight exposure [73,74] and living at high altitudes [75,76].

Genetic factors have also been identified as contributing to the pathogenesis and development of cataracts. There are also cataracts that form from a single gene: monogenetic cataracts. A study conducted with monozygotic and dizygotic twins found that genetics contributed to almost 50% of cataracts’ variation and severity. The gene which is most consistently associated with age-related cataracts is GALK1, which has been seen in Asians and Caucasians [77,78,79]. EPHA2 is also associated with the development of age-related cataracts within patients from Pakistan, Western Europe, northern Italy, China, Britain and Australia [77,78,79]. Similar to PXF, there are genes associated with protection against age-related cataracts, such with the gene SPARC [80].

### 1.3. Ocular Manifestations of Both PXF and Cataract

Patients with PXF are generally asymptomatic in the beginning with little vision loss or eye pain [81]. It is usually not determined that a patient has PXF until a secondary outcome is observed, when vision can become impaired, such as PXF co-occurring with glaucoma [82]. Many cases of PXF are diagnosed during an eye examination, because PXF is considered a risk factor for just not glaucoma, but cataracts as well [83,84]. PXF, on its own, can become problematic if it blocks the trabecular meshwork and leads to elevated ocular pressure [81,82,83,84]. PXF can be missed in 20% of eyes that have not been dilated during an ophthalmic exam [82]. Pseudoexfoliation glaucoma (PXG) is present in 15% to 30% of the population with PXF, and is different from primary angle glaucoma in that the IOP elevates more quickly [5]. PXF is present in 30–40% of the population with cataracts. Currently, the molecular pathobiology associated with this clinical finding is unknown, but current research suggests oxidative stress [85,86,87,88].

### 1.4. Underserved Populations and Eye Conditions

PXF and cataract prevalence as well as other blinding eye diseases differ by continent, country and regions within the country and socio-economic status of the communities. Underserved and isolated populations have unique paradigms for disease prevention and risk [34,89]. These populations may experience more significant health disparities due to decreased access to education, nutritional food sources, community and social support, safe neighborhoods, and quality healthcare [90,91,92]. Guatemala was able to eliminate onchocerciasis, or river blindness [93]. Although this is a significant public health achievement, other blinding eye diseases still remain a substantial public health issue. To date, only two studies have been conducted with respect to PXF in Guatemala [94,95,96]. Other studies have reported that cataracts are the leading cause of blindness within Guatemala, because barriers to intervention include the cost of surgery, fear of surgery, and lack of knowledge about cataract surgery [96].

There is a lack of information on the unique characteristics of geographically isolated communities within Guatemala in terms of chronic disease prevalence in general, and moreover, in terms of the prevalence of eye disease. Factors that may drive ophthalmic disease prevalence include demographic, environmental, and biomarker drivers of disease risk [97,98,99]. Within Guatemala, indigenous populations, such as the Maya, disproportionally experience health disparities [100,101,102]. The public health services provided to indigenous populations of Latin America, including the Maya, are below the standard of care in general [103]. In addition, the indigenous population, comprising mainly the Mayan population, has a thirteen-year lower life-expectancy than non-indigenous populations in Guatemala [100]. This may, in part, be due to the geographic isolation of the Mayans and access to preventative and health care services, including eye care services [100,104]. Therefore, to better understand the prevalence of blinding eye disease and risk factors driving eye disease in this specific population, we ascertained and recruited an observational cohort of individuals seeking eye care in Salama, Guatemala, at the Lions Eye hospital to identify clinical, demographic, environmental and/or genetic risk factors for blinding eye disease in an effort to inform targeted and effective screening strategies to achieve better improved health care distribution. The overall goal of this study was to determine ocular health needs within this population and any possible modifiable risk factors contributing to disease pathophysiology.

## 2. Materials and Methods

### 2.1. Mayan Study Cohort

The study protocol was reviewed and approved by the Institutional Review Board at the University of Utah (University of Utah IRB#52879). This study conformed to the tenets of the Declaration of Helsinki. The study was also locally reviewed for ethical risk and approved by the Salama Lions Eye Club Hospital in Guatemala, as well as the Minister of Public Health and Social Assistance in Guatemala. The study ensured that the research adhered to local laws and cultural beliefs. Study participants were enrolled in this study after receiving detailed information about the research project and giving written informed consent. The study team visited the Salama Lions Eye Club Hospital in Guatemala. The study team explained the importance and benefits of the study to the Salama Lions Eye Club Hospital leaders. The study team, in conjunction with the Salama Lions Eye Club Hospital, utilized an outreach eye camp in which participants were seen. In total, we recruited 126 participants, 18 years and older, for this study. These participants were willing to participate in the study as they were being seen at the outreach eye camp. We did not deny anyone access to care; thus, participants still received care even if they did not participate in the study. The study was able to recruit those who were seven years and older, but only included adults. Each study participant received a comprehensive eye examination performed by a board-certified ophthalmologist. Participants were also asked to provide a blood sample for a genome-wide association study with disease status.

Furthermore, participants were asked to complete a structured core baseline interview for a standardized epidemiological questionnaire administered by the study team member in conjunction with an interpreter [90,105,106,107,108]. Interpreters provided detailed information and assisted with the consent form and questionnaire in the participants’ native dialect (Appendix A). Interpreters were from the University of San Carlos linguistic program. We employed a detailed standardized structured core baseline interview for an epidemiological questionnaire that assessed clinical, demographic (including indigenous culture), and environmental risk factors. Board-certified ophthalmologists assessed phenotypes, while the epidemiological questionnaire was reported by the study participants in conjunction with an interpreter when necessary. The data collected were stored in a secure password-protected database.

### 2.2. Phenotyping

All study participants received a comprehensive ophthalmic examination. Our study included four board-certified ophthalmologists who completed the dilated ophthalmic examination. The examinations included a magnified assessment of the anterior and indirect ophthalmoscopy of the dilated posterior segment, as previously described [109,110]. Mydriatic fundus images were obtained from both eyes of all study participants. The digital retina photos were evaluated by board-certified ophthalmologists. Therefore, each study participant received a clinical-level evaluation for the presence or absence of blinding eye disease, and phenotypes were not self-reported.

### 2.3. Epidemiological Analysis

Factors associated with eye disease outcomes within our study participants were evaluated to determine the degree of any statistical associations. Participants were only included in the epidemiological analysis if the data were more than sixty percent complete. We conducted data imputation in the statistical programming language R [111]. In R, the multivariate imputation was performed with the chained equations (MICE) package, which creates multiple imputations for missing data, including continuous, binary, unordered, and ordered categorical data [111,112]. Each of the incomplete variables is imputed by a separate model using the fully conditional specification method [111,112]. There were variables included in our analysis for which we implemented the MICE package to impute (Appendix B, Table A1). Next, univariate (unadjusted model) and multivariate analyses (adjusted model) were conducted for eye disease outcomes. The three eye diseases/conditions examined further as outcomes of interest were cataracts, PXF, and having both cataracts and PXF. Those that had the observed event or disease outcome (cases of either PXF, cataracts or both) were compared to those that did not have the observed event or the controls. These were chosen as the outcomes of interest because, as will be discussed, they were the most prevalent blinding eye disease/conditions within the study population. We evaluated clinical, demographic, and environmental factors associated with the eye disease/conditions of interest. The demographic factors that were evaluated within the univariate analyses included age (continuous), sex (male vs. female), marital status (married vs. non-married), and smoking status (ever smoker vs. never smoker). The clinical factors evaluated within the univariate analyses included diabetes mellitus, hypertension, elevated cholesterol, asthma, myocardial infarction, other heart conditions, and weight. The environmental factors evaluated within the univariate analyses were elevation, low-resource department, and outside working conditions. After conducting each of the univariate analyses, only statistically significant factors at *p* < 0.10 were included in the multivariate model [108]. Factors that remained statically significant at *p* < 0.05 were considered statistically significantly associated with the eye disease/condition of interest [108]. In observing if there was an association of PXF on having a cataract diagnosis, we conducted a univariate analysis. We then included age and sex in the multivariate model to determine if an association remained significant at *p* < 0.05.

### 2.4. Genetic Ascertainment

Blood samples were taken from study participants and processed for the genetic analysis (Appendix C). Two 10 mL tubes of blood were collected for each of the study participants. Leukocyte DNA was purified using standard phenol–chloroform extraction methods, the DNAzol (Invitrogen) extraction protocol, or the QIAamp DNA Blood Maxi Kit (Qiagen), as previously reported [109]. DNA was extracted from the peripheral blood leukocytes from the blood samples, and candidate single nucleotide polymorphisms (SNPs) were genotyped [105]. The cohort of 126 study participants was genotyped using Illumina’s HumanOmni2.5-8 (Omni2.5) BeadChip v1.2 (San Diego, CA, USA). SNP data cleaning and analysis were performed using PLINK version 1.9 [113,114]. Data cleaning was performed following standard protocols [115]. Specifically, quality control measures included evaluating SNPs and individuals’ missingness, sex discrepancy, minor allele frequency (MAF), and Hardy–Weinberg equilibrium (HWE) [115]. Individuals missing more than 2% of SNPs were removed from the analyses [115]. Additionally, SNPs missing in more than 2% of the samples were removed from the analyses [115]. Our MAF threshold for inclusion was 0.05, and HWE was set at *p* < 1^−10^ [115,116]. After data cleaning, we performed logistic regression using PLINK version 1.9 to examine the phenotypes of interest within our study populations [115,117]. We examined three phenotypes: cataracts, PXF, and both. The individuals with these phenotypes were the cases, whereas those without the phenotypes were the controls. Additionally, we conducted four separate logistic regression models for each outcome of interest. We adjusted for age, sex, both age and sex, and no covariates in these models. Minor allele frequencies in the cases and the controls were calculated for the SNPs that were statistically significant for the outcomes of interest. We implemented sequence kernel association testing analysis (SKAT) using the SKAT package (v2.0.1) in R (v4.0.2) (R Foundation for Statical Computing, Vienna, Austria). For each gene harboring a significant SNP, we looked at all SNPs lying within the gene, as well as those within 500 bp upstream and downstream of the gene. We included any of these SNPs that had an unadjusted (*p* < 0.05) in our SNP sets for SKAT analysis for the gene. This gave us eight SNP sets to use in our SKAT analysis cataracts, and one for PXF. We performed SKAT analysis for eight genes testing for association with cataracts, and one gene testing for association with PXF.

### 2.5. Bioinformatics

We utilized the gene databases National Center for Biotechnology Information Phenotype-Genotype Integrator (NCBI PhenGenI) and Ensembl release 10 to determine if any of the genes that were identified in our genome-wide multivariate logistic regression analysis was associated with cataracts, PXF, or both. Furthermore, we utilized the peer-reviewed pathway database Reactome, conducted a pathway analysis using the SNPs that were associated with cataracts at genome-wide significance (*p* < 5 × 10^−8^) and PXF at genome-wide significance (*p* < 1 × 10^−5^).

## 3. Results

### 3.1. Eye Disease and Conditions

In our study, 126 study participants received a comprehensive ophthalmic examination. Our population’s average age that received comprehensive ophthalmic examinations was 65.2, and males made up 46%. We found that almost one-quarter of our study participants had a diagnosis of PXF and more than half had a diagnosis of cataracts. In individuals with cataracts, 28 of them also had PXF. In terms of glaucoma, 4% of the study participants had a diagnosis of glaucoma. Two individuals had both PXF and glaucoma. There were cases of other eye conditions, including diabetic retinopathy, age-related macular degeneration, peripapillary atrophy, and pterygium in our study population (Table 1).

When observing those with an eye diagnosis of PXF, cataracts, glaucoma, or PXF with glaucoma or cataracts, males made up less than half of the population with a diagnosis, except in the case of a diagnosis of cataracts. All participants that were diagnosed with both PXF and glaucoma were female. Not surprisingly, all participants with a diagnosis of glaucoma, cataracts, PXF, or a combination, were over the age of 40 (Table 2).

### 3.2. Epidemiological Analysis and Blinding Eye Disease

After excluding patients with incomplete data, 121 study participants were included in the epidemiological analysis for association with blinding eye disease. There were slightly more females than males in our epidemiological analysis (54.44%). Our study participants’ average age within the epidemiological analysis was about 65, with age ranging from 21.6 to 96.1 years of age (Table 3).

In the univariate analysis for associated risk factors for PXF at *p* < 0.10, age of the study participant, heart attack and other heart conditions were associated with the outcome. For every year increase in age, there were 1.071 greater odds for a diagnosis of PXF. Those who had a heart attack had 4.212 greater odds for a diagnosis of PXF as compared to those that had not had a heart attack. Those that had another heart condition had 3.417 greater odds for a diagnosis of PXF as compared to those that did not have a diagnosis—associated risk factors at *p* < 0.10 for a diagnosis of cataracts included both the age of the study participant and living in one of the ten lowest-resourced departments in Guatemala. For every year increase in age, there were 1.111 greater odds for a diagnosis of cataracts. Those living in one of the low-resourced departments in Guatemala had 2.659 greater odds of having a diagnosis of cataracts than those that did not. Looking at risk factors for both PXF and cataracts, combined age and having a heart attack were statistically associated at *p* < 0.10. For every year increase in the study participant’s age, there were 1.075 greater odds for PXF and cataracts combined. Those who had a heart attack had 5.253 greater odds of developing PXF and cataracts than those who had not (Table 4).

In the multivariate analysis of associated risk factors for PXF in our study population, we found that age was the only factor that remained significant at *p* < 0.05. Both the factors of having a heart attack or another heart condition were no longer statistically associated with having a diagnosis of PXF in the multivariate model at *p* < 0.05. For every year increase in age, there were 1.068 greater odds of PXF in our study population. In terms of cataracts, both age and living in a lower resourced department remained statistically significantly associated with cataracts in the multivariate model at *p* < 0.05. For every year increase in age, there were 1.119 greater odds of a cataract diagnosis. Those living in a lower-resourced division had 3.214 greater odds of having a cataract diagnosis compared to those that did not live in one of the ten departments with the lowest amount of resources. Observing the outcome of having a diagnosis of both PXF and cataracts, only age remained statically significant at *p* < 0.05. There were 1.069 greater odds of having both PXF and cataracts in our study population for every year increase in age. Having a heart attack was no longer statically significant with a diagnosis of both PXF and cataracts at *p* < 0.05 (Table 5).

We sought to explore if PXF was associated with an outcome of cataracts in our study population. We found that even after adjusting for age and sex, PXF was still significantly associated with a diagnosis of cataracts in our study population at (*p* < 0.05). Those with a diagnosis of PXF had 5.866-fold increased odds of having a diagnosis of cataracts as compared to those that did not have a diagnosis of PXF after adjusting for both age and sex in the model at *p* < 0.05 (Table 6).

### 3.3. Genetic Analysis

After completing all quality control measures, there were 1,093,747 SNPs for our association study within 123 samples. There was almost 48% of the original SNPs that were included before quality control measures were implemented, with three individuals dropped after the quality control measures (one for missingness and two for sex-discrepancies). We identified 33 SNPs associated with a diagnosis of cataracts at genome-wide significance after adjusting for age and sex (*p* < 5 × 10^−8^). These SNPs were found within 13 genes: CFAP74, FRMD4B, NAALADL2, LOC105377670, ACSL1, LOC101927668, LINC00968, LOC101929415, LOC107984170, CTNNA3, PRCP, LOC105369844, ZNF423. We found one SNP associated with a diagnosis of PXF after adjusting for sex with nominal significance (*p* < 1 × 10^−5^). This SNP lay within the gene KSR2. We did not find any SNPs significantly associated with having a diagnosis of both cataracts and PXF (Table 7).

We performed SNP-set analysis using SKAT in R to analyze SNP sets in eight genes for associations with cataracts and one gene for associations with PXF. The SNP sets for the cataract analyses contained between 8 and 40 SNPs, whereas the PXF set was made up of 26 SNPs. For all analyses, the individual SNP initially found to be associated with disease was more significant than the SNP set (Table 8).

### 3.4. Bioinformatics

Utilizing both PhenGenI and Ensembl release 10, the SNPs within our genome-wide association study analysis did not uncover any similar gene associations with PXF, cataracts, or both, as previously reported. Using the peer-reviewed pathway database Reactome, and performing pathway analysis, including interactions, we found that four genes associated with cataracts, ZNF423, PRCP, ACSL1, and CTNNA3, were involved with DNA replication, signal transduction, metabolism, and the immune system top-level pathways. Using the same pathway database, we found that for the PXF-associated gene KSR2, Disease, Signal Transduction, Metabolism, Gene Expression and Cellular Response to external stimuli were the top-level pathways (Table 9).

## 4. Discussion

### 4.1. Eye Disease Prevalence in Underserved Populations

Guatemala has the largest proportionately indigenous population in Latin America, with more than 60% of the population being native to the region [118]. Within Guatemala, the majority of indigenous people are of Mayan descent [119]. Little is known about blinding eye disease prevalence in Guatemala, and there have been only two population-based studies. One considered participants from all 22 departments in Guatemala, equivalent to geographic regions within the country, divided administratively and politically [120,121]. Few studies to date have examined PXF, as well as PXF and cataracts in Guatemala, specifically within the rural Mayan population [94,95]. This is the first study to report both epidemiological and genetic factors that influence PXF and cataract risk within a Mayan cohort in Guatemala. In terms of disease prevalence, we found that 24.6% had PXF, 54.76% had age-related cataracts, and 22.2% had both PXF and age-related cataracts. With respect to epidemiological factors, we corroborated that age and fewer resources were significantly associated with cataracts [52], and separately, age with PXF [10,11,12]. Although we examined diabetes, elevation, and smoking, we did not find these factors to be associated with a risk of any ocular disease examined in this population. These factors have been found to be associated with PXF and/or cataracts in other studies; thus, additional studies may want to consider these variables.

A 2004 study focused only on diagnosing age-related cataracts. The study included 4806 participants from four departments, aged 50 years and older, who received examinations [120]. This hospital-based study found that 66.1% of the study participants had blindness due to cataracts [120]. We recruited all-comers 18 years and older, which may be why we found a lower prevalence of age-related cataracts because we did not exclude anybody based on disease status.

In 2015, the National Survey of Blindness and Visual Impairment in Guatemala found that 77.6% of the population (*n* = 3760 individuals 50 years and older) within the entire country, who were seen at their nearest medical unit, had blindness due to cataracts [121]. PXF was not explicitly mentioned within this study population, either separately or coexisting with cataracts. It is essential to highlight that both previous studies only included individuals 50 years of age and older. Our study included those 21.6–96.1 years old and found eye disease in those under the age of 50. However, our epidemiological factors for the risk of age-related cataracts were similar to those previously reported. If possible, including individuals from a wide age range would likely capture systemic conditions that may be risk factors for eye disease, such as hypertension and diabetes, which can occur earlier in life, especially in underserved populations.

We found that almost 24% of our homogenous geographically isolated population from the Salama Lions Eye Club Hospital (2 h and 45 min away from Guatemala City) had a diagnosis of PXF. In 2016, the Unit Glaucoma Clinic National of Oftalmología (CGUNO) in Ciudad of Guatemala, in Guatemala City, reported that men had a higher frequency of PXF compared to women; however, we did not find this [94]. This hospital-based study found that about 5% of patients aged over 45 years old had PXF, and about 4% in the total general population of all ages studied in Guatemala [94]. Guatemala City is the capital of Guatemala; the Salama Lions Club Eye Hospital is located in the department of Baja Verapaz. Baja Verapaz is considered to have fewer resources, which might be the reason behind the greater prevalence of PXF in addition to the potential differences in demographic and genetic backgrounds between the two departments [122]. A retrospective study, conducted at the Hospital de la Familia in Guatemala in the capital city, only included individuals with a prior diagnosis of cataracts [95]. They found that PXF was present in 15% of their study population, who already had cataracts [95]. Almost 23% of our study population had PXF with cataracts [95]. This difference in prevalence might be due to location. Salama is located in a department with fewer medical resources; San Marcos is the capital of its department with the same name [122]. Clinically, PXF has been associated with the formation of cataracts, which has been seen in populations living in Australia, Germany, and Iceland. Similar to others, we also confirmed this association within our study participation even after adjusting for both age and sex [4,83,123,124]. These groups showed that 1.67–45% had a prevalence of PXF and cataracts. Patients from lower resource areas, presenting with both PXF and cataracts may have additional challenges to recovery, because they may be more likely to have complications while receiving cataract surgery and post-cataract surgery. Knowing a priori what the unique risks are for having both PXF and cataracts would help to ensure that these individuals know the risks associated with pre-operative and post-operative cataract surgical care [81,123,125]. This is in line with previous research that has shown a high prevalence of cataracts in areas with low resources [81,123,125]. Furthermore, clinically, PXF has also been significantly associated with the development of open-angle glaucoma [16]. Although we did not have many cases of PXF and glaucoma (1.6%), those presenting with PXF are at risk of developing glaucoma. If glaucoma is left untreated, it can cause blindness. Similarly, to cataracts, those living in low-resourced areas may have greater difficulty managing glaucoma due to additional challenges including the access to care. In Guatemala, access to eye care services is limited and may not be easily accessible to all. Guatemala only has 13 ophthalmologists per million, compared to 60 ophthalmologists per million in the United States [126]. Ophthalmologists may be more concentrated in the cities in Guatemala, rather than rural areas. This can make it difficult for those in Guatemala who are already being disproportionally burdened by health disparities to obtain the needed care due to complications from having coexisting eye diseases.

### 4.2. Findings from Epidemiological Factors

Age is a risk factor for both PXF and cataracts and was confirmed within our study population for all disease outcomes [10,11,12]. We determined, using a cross-sectional study power calculation, that we had the power to detect an association with PXF, cataracts, and both PXF and cataracts with a 95% confidence interval, with powers of 93.63%, 99.93%, and 96.65%, respectively [127,128,129]. The population in Guatemala continues to grow older than in previous years, and may experience greater health inequity than the younger population [130,131]. The average life expectancy for those residing in Guatemala is now around 70 years old, compared to about 50 years old in 1970 [118]. Although greater life-expectancy is a public health accomplishment, it is crucial to help ensure that a support system is in place for healthy aging and to address health inequalities, which includes disease prevention in old age. Policies and interventions can be implemented to ensure healthy aging in communities with fewer resources [131].

The aging population may experience a greater prevalence of chronic disease, increasing their risk for developing secondary health outcomes, such as blinding eye disease [118,132]. A recent cross-sectional study, conducted by the Penn Center for Global Health, included 400 study participants [118]. The study found in the rural indigenous community of Atitlán, Guatemala, that diabetes was more significant than previously reported, with an almost 14% prevalence in 2018 compared to the previous 8.4% in 2003 [118]. We found that 13.2% of the indigenous Mayan population had diabetes, which is similar. We also found that advanced age was associated with diabetes. As previously discussed, diabetes is a risk factor often associated with cataracts [50,133]. Despite a prevalence of 13.2% for diabetes, we only observed one case of diabetic retinopathy. To date, the prevalence of diabetic retinopathy in a rural population in Escuintla, Guatemala, has been found to be about 6%, despite the public health epidemic of type II diabetes in Guatemala and its indigenous population [134]. With the increase in the public health epidemic of diabetes type II, there may also be an increase in cataract prevalence Guatemala [118,133]. Countries with low resource levels are disproportionally burdened by type II diabetes compared to countries with more significant resources [135,136]. Addressing public health issues that can impact the eye is essential, especially in underserved populations with less access to preventative and corrective care. Individuals with diabetes should be informed of their risk for developing blinding eye diseases, to include cataracts and as well as diabetic retinopathy [53,133,137,138]. Studies have supported an association of developing and worsening of diabetic retinopathy after receiving cataract surgery in those with diabetes; therefore, screening implications and eye health education should be implemented primarily in those with this chronic disease [139,140].

Our findings showed that those who lived in a low-resourced department based on their epidemiological questionnaire were more likely to have cataracts than those who did not, which is in line with previous reports [56,61]. These departments include the departments of Alta Verapaz, Sololá, Totonicapán, Quiché, Huehuetenango, Chiquimula, Jalapa, Baja Verapaz, Chimaltenango, and Suchitepéquez. In fact, these departments have been identified as having the most significant incidence of poverty, as confirmed by The National Employment and Income Survey of 2014 in Guatemala [122]. This survey also found that indigenous populations had a greater poverty incidence, with a rate of 79.2% compared to non-indigenous populations with a poverty rate of 46.6% [122]. Guatemala has the fifth most impoverished economy in Latin America and the Caribbean [141]. The COVID-19 pandemic has negatively impacted the economy in Guatemala, and it is expected that the poverty rate will increase due to the global pandemic. [141]. This will further impact health inequalities, including access to preventative and medical care [142]. Healthcare services and prevention methods concerning eye health should be tailored for the individual indigenous populations of Guatemala to create the change needed to address health disparities. Otherwise, broad-level planning may not effectively address these inequalities which are more prevalent now, during a global pandemic, than ever before.

### 4.3. Findings from Genetic Factors

Along with epidemiologic factors, we also found genetic factors to be associated with disease outcomes in participants. Our study demonstrated that rs895471 KSR2 was nominally significantly associated with reduction in risk of PXF. We also found that 33 SNPs in 13 genes were significantly associated with a risk of age-related cataracts at genome-wide significance (10^−8^). Some of these genes associated with age-related cataracts include FRMD4B, NAALADL2, LOC105377670, LINC00968, LOC101929415, CTNNA3, LOC107984170, PRCP, and ZNF423, whereas others with a reduced risk include CFAP74, ACSL1, LOC101927668, LOC105369844. These genes and these SNPs are unique to PXF, and separately, age-related cataracts in the Mayan population of Guatemala have not previously been reported by other groups and/or the National Center for Biotechnology Information Phenotype-Genotype Integrator (NCBI PhenGenI) [38,43,44,46,75,77,143]. As previously published, we did not find any significant associations with PXF and cataract combined. Similarly, we interrogated Ensembl release 10 (http://useast.ensembl.org/index.html; accessed on 10 February 2021), and additionally confirmed that these same SNPs and genes were not associated with PXF and age-related cataract disease outcomes [144]. Utilizing PhenGenI, the gene found to be associated with PXF in our GWAS analysis was KSR2 rs895471, and the connector enhancer of this gene (CNKSR2) was found to be associated with age-related macular degeneration [143]. This was confirmed within the National Eye Institute (NEI) Age-Related Eye Disease Study in a European population (dbGaP Study Accession: phs000001.v3.p1) [145].

We observed three cases of AMD (including one case of dry-AMD). We found that all of our participants with AMD were heterozygous for KSR2 rs895471 (A > G), and variation in KSR2 has been associated with obesity, insulin resistance, and impaired cellular fuel oxidation [146]. We observed that the frequency of the minor allele G at KSR2 rs895471 (A > G) was higher in our diabetic study participants compared to our non-diabetic study participants (G = 0.441 vs. G = 0.393, respectively), although not statistically significant (*p* < 0.05).

Utilizing the pathway database Reactome (https://reactome.org; accessed 10 February 2021), we conducted an analysis using the SNPs that were associated with cataracts at genome-wide significance (*p* < 5 × 10^−8^) [147]. The pathway analysis, including interactions, showed that four genes (ZNF423, PRCP, ACSL1, and CTNNA3) were involved in DNA replication, signal transduction, metabolism, and the immune system [147]. Metabolic diseases have been associated with the formation of cataracts [148,149]. Components of metabolic disease that put individuals at a greater risk for cataract formation included both diabetes and hypertension. An example of this association be found in a population-based study conducted in Singapore, which included 2794 Malay adults aged 40 to 80 years old for analysis [149]. Of the study participants, 48.3% were male and 42.2% of the study population had metabolic syndrome [149]. The study found that the prevalence of cataracts increased with metabolic syndrome components, hypertension and diabetes [149]. We found that variants in the genes associated with age-related cataracts, including NAALADL2 and ACSL1, functioned in the metabolism pathway. This is similar to what others have reported when observing GALK1 and cataracts in the metabolism pathway. [147]

We also interrogated the Reactome database to conduct a pathway analysis for the nominally associated KSR2. (*p* < 1 × 10^−5^) with PXF. We found that Disease, Signal Transduction, Metabolism, Gene Expression and Cellular Response to external stimuli were the top-level pathways [147]. The “disease pathway” is similar to the disease pathway associated with the gene SOD2, previously associated with PXF [147]. Both electron microscopy and immunohistology studies demonstrate that the white dandruff-like exfoliation material on the lens capsule of the eye in individuals with PXF is the outcome of abnormal extracellular matrix material metabolism [150]. Moreover, gene expression studies have found an association with PXF and cellular metabolism, specifically in pseudoexfoliation glaucoma, demonstrating that differentially regulated genes are involved with extracellular matrix structure and metabolism as well as responses to stress and inflammation [151,152,153]. This includes the gene which is most often associated with PXF, LOXL1 [152]. Studying the genetics and epidemiological factors in this unique population may have broader implications for understanding complex diseases in other populations around the world. This population is considered geographically isolated; therefore, there may be few differences in their lifestyle factors, and any identified genes from this study may likely be truly associated with the disease [154]. These identified risk-associated genetic variants can help identify individuals living in Guatemala who may be at greater risk of developing the disease. By identifying these individuals, preventative care measures can be implemented, as well as the distribution of appropriate resources to diagnose, manage and potentially treat these conditions. Although genetic tests can be difficult to access in low-resource countries, there have been efforts to make community genetics programs more accessible, in particular for the treatment and management of cataracts. Our identified genes could be utilized to contribute to this care once implemented [155].

### 4.4. Future Directions

Rural, isolated populations, even with a small study sample size, are essential populations to include in research, because if they are ignored, health disparities will only further increase [90,105]. Future research is needed to validate the genetic findings based on leukocyte DNA from blood. One way may be to study the ocular lens tissues from these patients in order to confirm our genetic association studies at the gene expression or protein level, which could attribute function to these identified risk variants. We utilized an agnostic genetic approach to determine if there were associations with PXF, cataracts, or a co-existing diagnosis. Future research may take a candidate gene-based approach to determine if the genes and SNPs relevant in our study are associated with these disease states in other populations. This is further supported by the pathway analyses conducted in the overlap of top-level pathways between the genes identified previously for cataracts and PXF as well as the associations of these diseases with these pathways. There are current health disparities that exist between different population sectors within the country of Guatemala. Therefore, research efforts should concentrate on studies that will develop healthcare services and prevention methods to address these health disparities to achieve health equity in these populations. Research is further needed to address health disparities for ingenious populations in Guatemala that are more greatly impacted by poverty and health inequity. Our study did not exclusively observe different Maya cultural groups; therefore, future studies may identify specific wants and needs pertaining to eye health and eye research within the more than 20 different Guatemala Mayan communities that differ by both language and culture [156]. Our previous research within the American Indian tribes of the Intermountain West has shown that there are differences in the prevalence of blinding eye disease within the same state at the tribal level; therefore, this research is essential to determine differences that may occur between Mayan communities in Guatemala [90,105].

## 5. Conclusions

Our study supports the importance of understanding blinding eye disease in geographically isolated populations. Cataracts are known to be more prevalent and under-cared for in low-resource countries. Individual communities with lower resources in these countries may be more significantly impacted. Both healthcare services and preventative care methods developed at a broad level may not create the change needed to address health disparities between different communities in different geographic locations. Our GWAS analysis results may impact future studies within this population, focusing on genetic components of associated eye disease. We found that KSR2 was nominally associated with PXF and 13 novel genes associated with cataracts at a significant genome-wide level. This study can inform the prevalence of genetic epidemiological risk factors associated with PXF and age-related cataracts within this population. It may have more significant implications for the screening of blinding diseases in underserved populations in general. This research should help to inform service delivery for these populations and allow those planning and delivering relevant health services and policymakers to tailor those services to achieve maximum access, relevance, and utilization by the members of the individual communities in Guatemala.

## Figures and Tables

**Table 1:** Demographics in the eye examination study participants.

Demographics	Total Eye Exam Participants (*n* = 126)
PXF	31 (24.6%)
Cataracts	69 (54.8%)
Glaucoma	5 (4.0%)
PXF with Glaucoma	2 (1.6%)
PXF with Cataracts	28 (22.2%)
Diabetic Retinopathy	1 (0.8%)
Age-Related Macular Degeneration	2 (1.6%)
Dry AMD—AREDS3	1 (0.8%)
Peripapillary Atrophy	1 (0.8%)
Pterygium	4 (3.2%)
Age (range)	65.2 (21.6–96.1)
Male Sex	58 (46.0%)

Dry AMD—AREDS3 is Dry Age-Related Macular Degeneration Age Related Eye Disease Study.

**Table 2:** Age and sex of PXF, cataracts and glaucoma participants.

Diagnosis	Age (Range)	Male Sex
PXF (*n* = 31)	74.3 (59.9–90.4)	14 (45.2%)
Glaucoma (*n* = 5)	66.4 (53.4–79.4)	2 (40%)
Cataracts (*n* = 69)	73.4 (42.2–96.1)	35 (50.7%)
PXF + Glaucoma (*n* = 2)	64.6 (62.1–67.1)	0 (0%)
PXF + Cataracts (*n* = 28)	74.8 (62.1–90.4)	12 (42.9%)

**Table 3:** Demographic table for participants used in univariate and multivariate analyses.

Demographics	Total # of Participants in Adjusted Analyses (*n* = 121)
Age (range)	64.6 (21.6–96.1)
Gender	
Male	55 (45.45%)
Female	66 (54.55%)
Married *	62 (51.24%)
Smoker *	26 (21.49%)
Outdoor Working Condition *	45 (37.19%)
Diabetes *	16 (13.22%)
Hypertension *	31 (25.62%)
Heart Attack *	7 (5.79%)
Asthma *	8 (6.61%)
High Cholesterol *	9 (7.44%)
Other Heart Condition	7 (5.79%)
Elevation (range) in meters *	978.62 (7–2507)
Low-Resource Department	76 (62.81%)
Eye Disease/Condition	
PXF	29 (23.97%)
Cataracts	64 (52.89%)
PXF + Cataracts	26 (21.41%)

* Missing values for some participants—see Appendix A.

**Table 4:** Univariate analysis of epidemiological risk factors and eye conditions.

Characteristic	PXF OR (95% CI)	Cataracts OR (95% CI)	Cataracts + PXF OR (95% CI)
Age	1.071 (1.030–1.114)	1.111 (1.066–1.158)	1.075 (1.031–1.121)
Male Sex	1.161 (0.503–2.680)	1.478 (0.719–3.041)	1.037 (0.434–2.475)
Married	0.624 (0.250–1.561)	0.667 (0.269–1.651)	0.552 (0.215–1.418)
Smoker	0.597 (0.204–1.744)	0.859 (0.358–2.062)	0.683 (0.219–2.126)
Diabetes	1.250 (0.372–4.603)	2.300 (0.748–7.077)	1.478 (0.436–5.015)
HBP	1.222 (0.471–3.165)	1.680 (0.731–3.858)	1.266 (0.477–3.359)
High Cholesterol	0.450 (0.0044–4.603)	0.595 (0.158–2.236)	0.834 (0.116–6.011)
Asthma	2.096 (0.398–11.024)	2.155 (0.665–6.970)	5.08 × 10^−8^ (0-Inf.)
Heart Attack	4.212 (0.885–20.042)	8.237 × 10^5^ (0-inf)	5.253 (1.089–25.332)
Other Heart Condition	3.417 (0.848–13.768)	2.223 (0.601–8.296)	2.483 (0.309–19.982)
Outside Working Conditions	1.204 (0.514–2.822)	1.685 (0.800–3.55)	1.257 (0.520–3.039)
Elevation	1.000 (0.999–1.000)	1.000 (0.999–1.000)	1.000 (0.999–1.001)
Weight	0.984 (0.962–1.008)	0.995 (0.981–1.009)	0.983 (0.960–1.007)
Low Resource	1.392 (0.423–4.579)	2.659 (1.068–6.662)	1.479 (0.414–5.277)

**Table 5:** Multivariate analysis of epidemiological risk factors and eye conditions.

Characteristic	PXF OR (95% CI)	Cataracts OR (95% CI)	PXF + Cataracts OR (95% CI)
Age	1.068 (1.024–1.113)	1.119 (1.071–1.169)	1.069 (1.025–1.116)
Male Sex	-	-	-
Married	-	-	-
Smoker	-	-	-
Diabetes	-	-	-
HBP	-	-	-
High Cholesterol	-	-	-
Asthma	-	-	-
Heart Attack	1.703 (0.316–9.170)	-	2.147 (0.417–11.058)
Other Heart Condition	3.091 (0.628–15.206)	-	-
Outside Working Conditions	-	-	-
Elevation	-	-	-
Weight	-	-	-
Low Resource	-	3.214 (1.096–9.424)	-

**Table 6:** PXF association with cataracts.

Univariate	Odds Ratio (95% CI)	*p*-Value
PXF	12.292 (3.493–43.249)	<0.001
Multivariate		
PXF	5.866 (1.559–22.078)	0.009
Age	1.102 (1.056–1.149)	< 0.001
Sex	1.072 (0.429–2.683)	0.881

**Table 7:** Associated SNPs with cataracts and PXF.

Phenotype	SNP	Gene	Alleles	Allele Frequency Cases	Allele Frequency Controls	*p*-Value
Cataracts	rs72636339	CFAP74	A > C	C = 0.1190	C = 0.1915	4.978 × 10^−8^
Cataracts	rs12492375	FRMD4B	C > T	T = 0.0714	T = 0.0532	4.109 × 10^−8^
Cataracts	rs6762603	FRMD4B	G > A	A = 0.0714	A = 0.0532	4.109 × 10^−8^
Cataracts	rs6790753	NAALADL2	C > T	C = 0.2540	C = 0.2128	4.449 × 10^−8^
Cataracts	rs4833139	Intergenic	G > A	A = 0.1905	A = 0.1170	4.800 × 10^−8^
Cataracts	rs79925560	LOC105377670	C > T	T = 0.2460	T = 0.2340	4.602 × 10^−8^
Cataracts	rs2412857	Intergenic	C > T	T = 0.2540	T = 0.2128	4.139 × 10^−8^
Cataracts	rs2100686	Intergenic	G > A	A = 0.4921	A = 0.3936	4.782 × 10^−8^
Cataracts	rs4862411	Intergenic	T > C	C = 0.3095	C = 0.4894	4.203 × 10^−8^
Cataracts	rs2292899	ACSL1	A > G	A = 0.3254	A = 0.5213	4.163 × 10^−8^
Cataracts	rs3815254	LOC101927668	T > C	C = 0.3968	C = 0.5319	4.153 × 10^−8^
Cataracts	rs13247232	LOC101927668	A > C	C = 0.4048	C = 0.5426	4.403 × 10^−8^
Cataracts	rs7789907	LOC101927668	C > A	A = 0.3968	A = 0.5000	4.196 × 10^−8^
Cataracts	rs11766281	LOC101927668	A > C	C = 0.4048	C = 0.5426	4.403 × 10^−8^
Cataracts	rs12700135	LOC101927668	A > G	G = 0.4048	G = 0.5319	3.955 × 10^−8^
Cataracts	rs6957312	LOC101927668	T > C	C = 0.4286	C = 0.5532	3.499 ×10^−8^
Cataracts	rs12667587	LOC101927668	C > T	T = 0.4365	T = 0.5638	4.387 ×10^−8^
Cataracts	rs6980441	LOC101927668	G > A	A = 0.4127	A = 0.5106	4.228 ×10^−8^
Cataracts	rs201711770	LOC101927668	G > A	A = 0.4365	A = 0.5532	4.734 ×10^−8^
Cataracts	rs3102070	Intergenic	G > A	T = 0.1270	T = 0.0745	4.515 × 10^−8^
Cataracts	rs71519459	LINC00968 and LOC101929415	T > C	C = 0.1587	C = 0.0957	4.343 × 10^−8^
Cataracts	rs1762004	LOC107984170	C > T	T = 0.1905	T = 0.1277	4.786 × 10^−8^
Cataracts	rs2489633	Intergenic	G > T	C = 0.1905	C = 0.1277	4.786 × 10^−8^
Cataracts	rs12250991	CTNNA3	T > C	C = 0.0952	C = 0.0319	4.826 × 10^−8^
Cataracts	rs12257968	CTNNA3	A > G	G = 0.0952	G = 0.0319	4.826 × 10^−8^
Cataracts	rs12251222	CTNNA3	G > A	A = 0.0952	A = 0.0319	4.147 × 10^−8^
Cataracts	rs1576479	Intergenic	A > G	G = 0.2222	G = 0.1596	4.724 × 10^−8^
Cataracts	rs1576480	Intergenic	T > C	C = 0.2143	C = 0.1383	4.411 × 10^−8^
Cataracts	rs7107322	PRCP	G > A	A = 0.2460	A = 0.1809	4.768 × 10^−8^
Cataracts	rs60376799	LOC105369844	A > G	G = 0.1587	G = 0.2660	4.480 × 10^−8^
Cataracts	rs16975803	Intergenic	C > T	T = 0.2222	T = 0.1596	4.903 × 10^−8^
Cataracts	rs184403357	ZNF423	A > T	T = 0.0952	T = 0.0425	4.381 × 10^−8^
Cataracts	rs2652921	Intergenic	G > A	G = 0.2619	G = 0.2065	0
PXF *	rs895471	KSR2	A > G	G = 0.1552	G = 0.500	8.628 × 10^−6^

* Sex adjusted only.

**Table 8:** Sequence kernel association testing analysis.

Phenotype	Gene	# of SNPs from GWAS	# of SNPs Used for SKAT Analysis	*p*-Value
Cataracts	CFAP74	12	12	0.16
Cataracts	FRMD4B	9	9	0.19
Cataracts	NAALADL2	8	8	0.033
Cataracts	ACSL1	10	10	0.0007
Cataracts	CTNNA3	40	40	0.30
Cataracts	PRCP	8	8	0.09
Cataracts	ZNF423	24	24	0.002
Cataracts	LOC101927668	12	12	0.10
PXF	KSR2	26	26	0.01

**Table 9:** Reactome pathway analysis.

Phenotype	Gene(s)	Top-Level Pathways
Cataracts	ZNF423 PRCP ACSL1 CTNNA3	DNA replication Signal transduction Metabolism Immune system
PXF	KSR2	Disease Signal Transduction Metabolism Gene Expression Cellular Response

## Data Availability

Data is available on request from the corresponding author.

## Appendix A

Documento de consentimiento y autorización

Título: Estudio genético y epidemiológico de enfermedades del ojo

Investigador principal: Margaret M. DeAngelis, Ph.D.

University of Utah

John A. Moran Eye Center

Salt Lake City, Utah

ANTECEDENTES

Se le pide que participe en un estudio de investigación. No tiene que participar en este estudio de investigación y si decide participar, puede cambiar de idea más adelante. Antes que decida, es importante que entienda por qué se está realizando la investigación y qué es lo que implicará.

Por favor, tome tiempo para leer detenidamente la siguiente información y háblelo con amigos y familiares si lo desea. Pregúntenos si hay algo que no está claro o si le gustaría tener más información. Tome tiempo para decidir si participará en este estudio de investigación.

El objetivo de este estudio es averiguar los genes y factores ambientales que podrían aumentar o disminuir el riesgo de desarrollar enfermedades que causan ceguera del ojo y enfermedades que están relacionadas con ellas.

No se conocen las causas exactas de estas enfermedades, pero es probable que tanto los factores genéticos como ambientales desempeñen un papel en su desarrollo. El determinar los genes y factores ambientales con las enfermedades del ojo puede ayudar a entender los mecanismos de la enfermedad subyacente y llevar a terapias potenciales.

La Dra. Margaret DeAngelis en el Departamento de Oftalmología y Ciencias Visuales, en la Universidad de Utah está realizando este estudio y lo auspicia en parte, los National Institutes of Health (NIH), Skaggs Foundation for Research y la Macular Degeneration Foundation.

PROCEDIMIENTOS DEL ESTUDIO

Si está de acuerdo en participar en este estudio de investigación, le pediremos que done una o más muestras de sangre de aproximadamente 30 a 50 mililitros. Se obtendrá ADN, ARN y proteína de la muestra de sangre para investigación genética. También le haremos preguntas sobre los factores de riesgo para ciertas enfermedades. En total tomará aproximadamente 60 minutos para completar.

Si es pertinente, se le pedirá que un oftalmólogo le examine los ojos. Es posible que ya se le hayan examinado los ojos como parte de un examen de rutina. Si ha tenido un examen de los ojos recientemente, no se le requerirá que se haga otro como parte del estudio. Si no ha tenido un examen de los ojos, el examen de los ojos que se le hará para este estudio incluirá la dilatación de la pupila con gotas para los ojos. Esto posiblemente le ocasionará visión borrosa y sensibilidad a la luz por unas horas. Evite manejar carro durante este tiempo. Como parte del examen, se tomarán fotografías de las retinas en sus ojos.

También se le pedirá que proporcione una muestra de enjuague bucal. Si está de acuerdo, se le darán 10 mililitros de enjuague bucal de venta libre que se enjuagará fuerte en la boca por 60 segundos. De ahí, se le pedirá que escupa la solución en un tubo esterilizado de colección. Si da una muestra de sangre o una muestra de enjuague bucal, estará dando una cantidad limitada de ADN para investigación genética continua para este estudio.

Si se obtuvo una muestra de tejido para patología, tal como una muestra de bazo o de médula ósea, nos gustaría usar la muestra para fines de investigación. Por favor, escriba sus iniciales en una de las siguientes líneas para indicar si da permiso para que usemos su muestra de tejido en este estudio de investigación.

_________ Sí, doy mi consentimiento para que se use mi muestra de tejido en el estudio de investigación.

_________ No, no quiero que mi muestra de tejido se use en este estudio de investigación.

Usted también tiene la opción de permitir que se genere una línea celular de su sangre para proporcionar un suministro renovable de ADN y de otros componentes celulares para investigación. Una línea celular es una muestra congelada de glóbulos blancos especialmente procesados de su sangre que nos permite crecer más glóbulos blancos y obtener más ADN y otros componentes celulares (por ejemplo, ARN y proteínas) de ellos según sea necesario para proyectos de investigación en el futuro.

Por favor, indique su decisión más adelante al escribir sus iniciales en una de las líneas:

_______ Sí, estoy de acuerdo en que se genere una línea celular de mi sangre para investigación de enfermedad de los ojos en el futuro.

_______ No, no deseo que se genere una línea celular de mi muestra de sangre.

_______ No es pertinente (si no se obtiene sangre)

Si nos enteramos que usted tiene una condición oftalmológica o de salud de la que no sabía anteriormente, le diremos al respecto.

ALMACENAMIENTO DE TEJIDO

Como parte de este estudio, nos gustaría poner parte de su sangre en el banco de tejido de la Dra. DeAngelis, para que otros investigadores puedan usarla en el futuro para investigación médica. La investigación que se haga en el futuro será específica a este estudio, lo cual se haría en su sangre y nos puede ayudar a aprender más sobre esta enfermedad. La Dra. DeAngelis, quien administra el banco de tejido en su laboratorio guardará toda la sangre o muestras de ADN. Las muestras de bazo y de médula ósea no se pondrán en un banco de tejido para investigación en el futuro.

Usted no tiene que participar en el banco de tejido para estar en la parte principal de este estudio. No importa lo que decida hacer, su decisión no afectará su atención médica.

Se obtendrá información que se identifique personalmente, incluso su nombre y apellido. No se le quitará la identificación a su muestra con el fin de que su muestra se devuelva correctamente. Cada muestra se ingresa en una base de datos y se le emite un número único de la muestra y lugar de almacenamiento en el congelador del laboratorio DeAngelis. Su “muestra maestra almacenada” tendrá su nombre y su apellido junto con el número único de identificación de muestra. Esta muestra maestra almacenada está almacenada en un congelador a -80C con candado y bajo llave y con un sistema de alarma las 24 horas. La base de datos electrónica que contiene esta información está protegida con contraseña y usuario y se mantiene para organizar el gran número de muestras que ingresan en el laboratorio DeAngelis, clasificada de acuerdo al diagnóstico de cada paciente. A todas las reservas funcionales, análisis realizados en el laboratorio y resultados subsecuentes se les quita la identificación, lo que significa que el nombre de los pacientes no está relacionado con el número único de identificación.

Además del ADN, ARN, suero y análisis epidemiológico también se mantienen en una base de datos segura personalizada para registro rápido de resultados. La base de datos se comparte entre los investigadores en el laboratorio DeAngelis y es accesible por medio de cualquier PC en el laboratorio. Está protegida con una contraseña para que cada investigador pueda ver los resultados que otros miembros del laboratorio ingresen, pero solo puede agregar o editar los resultados para los estudios, genes, variantes en los que el usuario está trabajando.

Si da permiso para que se guarden sus muestras para investigación sobre enfermedades que causan ceguera, que la Dra. DeAngelis haga en el futuro, el Comité de Revisión Institucional puede revisar y aprobar cada nuevo proyecto. El Comité de Revisión Institucional puede requerir que se comuniquen con usted para pedirle permiso antes de usar las muestras en un nuevo proyecto si se determina que se requiere un nuevo consentimiento para su protección.

Las muestras de tejido o de sangre que se obtengan de usted en esta investigación pueden ayudar en el desarrollo de un producto comercial de la Dra. DeAngelis o sus socios de investigación. No hay planes de darle compensación financiera si esto llegara a suceder.

Usted no recibirá resultados o hallazgos futuros.

Puede decidir retirar su muestra de sangre de este banco de tejido en cualquier momento. Se tendrá que comunicar con la Dra. DeAngelis al (801) 213–4052.

Por favor, seleccione una de las siguientes opciones:

________ Sí, se pueden guardar mis muestras para investigación sobre enfermedades que causan ceguera que realice la Dra. DeAngelis.

________ No, mis muestras se deben destruir al final de este proyecto de investigación.

Sub estudio de HII

Si está de acuerdo, también le preguntaremos sobre su historial médico familiar y revisaremos su carpeta médica para verificar las enfermedades oftalmológicas de interés y determinar si se le debería considerar para la prueba genética. También usaremos muestras de tejido y de sangre junto con su información del estudio principal e información clínica para determinar si hay una causa genética para la hipertensión intracraneal idiopática (HII). La HII es presión aumentada alrededor del cerebro que no la causa un tumor. Si no se trata, la HII puede causar pérdida de la visión.

Por favor, seleccione una de las siguientes opciones:

________ Sí, estoy de acuerdo en permitir que mis muestras e información se usen para el sub estudio de HII.

________ No, no quiero que se usen mis muestras ni información para el sub estudio de HII.

RIESGOS Y MOLESTIAS

Riesgos de la extracción de sangre:

Puede haber un poco de sangrado bajo la piel y un poco de sensibilidad en el lugar de la aguja. La infección de la piel es una posibilidad remota.

Riesgos del examen de los ojos:

Las gotas para dilatar los ojos que se usan rutinariamente en los exámenes de los ojos puede, en algunas personas, inducir glaucoma que requeriría que un oftalmólogo le dé tratamiento inmediato.

Un riesgo potencial es el abuso de confidencialidad, pero las protecciones, tales como el acceso restringido reducen este riesgo. Los archivos se almacenarán en archivos bajo llave en cuartos que no se están usando. Se codificarán y se protegerán con contraseña las computadoras portátiles y memorias que almacenen cualquier información protegida de salud.

BENEFICIOS POTENCIALES

No hay beneficios directos para usted por participar en este estudio de investigación. Sin embargo, si los genes u otros factores que tienen un papel en las enfermedades del estudio se descubren, pueden ayudar a los científicos a encontrar terapias para tratar estas enfermedades. Se debe enfatizar que aunque esta investigación sea exitosa, tomará varios años para que lleve a una nueva terapia.

PROCEDIMIENTOS ALTERNATIVOS

Usted no está bajo ninguna obligación de participar en este estudio de investigación y puede recibir atención médica para su condición sin participar en el estudio.

CONFIDENCIALIDAD

La información que se derive de este estudio se puede usar para fines de investigación, que pueden incluir publicación y enseñanza. Su información se tratará como confidencial en la medida en que la ley lo permita. Esto significa que le quitaremos la identificación a sus datos y su identidad se mantendrá confidencial. Sus datos se mantendrán seguros, en un área de acceso limitado a los miembros del equipo de investigación en el laboratorio DeAngelis.

La información digital se guardará en computadoras protegidas con contraseña. Solo se les permitirá el acceso a su información a aquellas personas que trabajan con este estudio.

Usted no recibirá los resultados de sus exámenes de sangre y los resultados no estarán disponibles para los empleadores, compañías de seguro, etc. para su privacidad y protección.

PERSONA CON QUIEN SE PUEDE COMUNICAR

Si tiene alguna pregunta, preocupación o queja o si siente que esta investigación le hizo daño, por favor, comuníquese con la Dra. Margaret DeAngelis, Departamento de Oftalmología y Ciencias Visuales en la Universidad de Utah, (801) 213–4052 durante horas hábiles. Si tiene una emergencia médica o preocupación después de horas hábiles, por favor, llame a la operadora del Hospital de la Universidad de Utah al (801) 581–2121. La operadora se puede comunicar con un médico de turno. Este número está disponible para llamar las 24 horas del día.

COMITÉ DE REVISIÓN INSTITUCIONAL: comuníquese con el Comité de Revisión Institucional (IRB, por sus siglas en inglés) si tiene preguntas referentes a sus derechos como participante de investigación. También, comuníquese con el IRB si tiene preguntas, quejas o preocupaciones que siente que no puede hablar con el investigador. Se puede comunicar con el IRB de la Universidad de Utah por teléfono al (801) 581–3655 o por correo electrónico a irb@hsc.utah.edu.

DEFENSOR DEL PARTICIPANTE DE INVESTIGACIÓN: también se puede comunicar con el Defensor del Participante de Investigación (RPA, por sus siglas en inglés) por teléfono al (801) 581–3803 o por correo electrónico a participant.advocate@hsc.utah.edu.

PARTICIPACIÓN VOLUNTARIA

Depende de usted decidir si participa o no en este estudio de investigación. Si decide participar, se le pedirá que firme este documento de consentimiento. Si decide participar, todavía tiene la libertad de retirarse en cualquier momento y sin dar ninguna razón. Esto no afectará la relación que tenga con el investigador o con su personal ni el estándar de atención que recibe.

Si decide retirarse del estudio, por favor, comuníquese con la Dra. Margaret DeAngelis, Department of Ophthalmology and Visual Sciences en la Universidad de Utah, (801) 213–4052, durante horas hábiles.

COSTOS Y COMPENSACIÓN PARA LOS PARTICIPANTES

No habrá ningún costo para usted por participar en este estudio. Los costos de cualquier procedimiento adicional que se haga debido a este estudio (tal como extracción de sangre) se cubrirán en la medida en que no los reembolse su compañía de seguro médico. Usted no recibirá ninguna compensación por su participación en este estudio.

NÚMERO DE PARTICIPANTES

Esperamos que se inscriban 10,000 o más participantes en este estudio. Este estudio se está realizando en la Universidad de Utah.

AUTORIZACIÓN PARA EL USO DE SU INFORMACIÓN PROTEGIDA DE SALUD

El firmar este documento significa que nos permite, a los investigadores en este estudio y a otros que trabajan con nosotros, usar la información sobre su salud para este estudio de investigación. Usted puede decidir si participa o no en este estudio de investigación. Sin embargo, con el fin de que participe tiene que firmar este documento de consentimiento y autorización. Esta es la información que usaremos:

- Su edad, género y raza
- Historial médico familiar
- Medicamentos o terapias actuales y anteriores
- Todos los otros exámenes y procedimientos que se realizarán en el estudio
- Cualquier otra información personal de salud que se obtendrá de otras fuentes para que se use en el registro de investigación, incluso el historial médico anterior, exámenes o registros de otros lugares

Otros que tendrán acceso a su información para este proyecto de investigación son el Comité de Revisión Institucional de la Universidad (el comité que supervisa la investigación que estudia a personas) y miembros autorizados de la Universidad de Utah quienes necesitan la información para cumplir con sus labores (por ejemplo: para brindar tratamiento, para garantizar la integridad de la investigación y para asuntos de contabilidad o facturación).

Si compartimos su información con alguien ajeno al University of Utah Health Sciences Center, no se identificará por nombre, número de seguro social, dirección, número de teléfono ni ninguna otra información que lo identifique directamente, a menos que la ley lo requiera.

En los registros y la información que se dé a conocer fuera del University of Utah Health Sciences Center, a su información se le asignará un número codificado único. Guardaremos la clave para el código en una computadora protegida con contraseñas o en archivos bajo llave. Destruiremos la clave para el código al final del estudio de investigación.

Usted puede suspender esta autorización, pero esto lo debe hacer por escrito. Tiene que darle su revocación ya sea en persona al investigador principal o a su personal o enviársela por correo a:

Dr. Margaret DeAngelis

University of Utah

John A. Moran Eye Center

65 Mario Capecchi Drive

Salt Lake City, UT 84132

Si usted suspende esta autorización, no podremos recopilar nueva información sobre usted y se le retirará del estudio de investigación. Sin embargo, podemos seguir usando la información que ya hayamos empezado a usar en nuestra investigación, según sea necesario para mantener la integridad de la misma.

Esta autorización no tiene fecha de vencimiento.

CONSENTIMIENTO:

Confirmo que he leído este documento de consentimiento y autorización y he tenido la oportunidad de hacer preguntas. Se me dará una copia firmada del documento de consentimiento y autorización para que la guarde.

Estoy de acuerdo en participar en este estudio de investigación y le autorizo a usted para que use y dé a conocer mi información de salud para este estudio, según me lo explicó en este documento.

Certifico que estoy familiarizado con los objetivos de este estudio y con sus posibles beneficios y riesgos. Estoy de acuerdo de manera voluntaria en participar como sujeto en el proyecto de investigación y entiendo que al firmar este documento de consentimiento estoy indicando ese acuerdo.

___________ _______________________________ _____________________________

Fecha Nombre del sujeto Firma

___________ _______________________________ _____________________________

Fecha Nombre de la persona que obtiene consentimiento Firma

## Appendix B

**Table A1:** Missing values that were imputed for univariate/multivariate epidemiological analysis.

Demographic	# of Missing Values Imputed
Married	28
Smoker	9
Outdoor Working Condition	2
Diabetes	1
Hypertension	2
Heart Attack	1
Asthma	28
High Cholesterol	3
Other Heart Condition	27
Elevation (range) in meters	17
Low-Resource Department	17

## Appendix C

Blood Sample Protocol

1. Study Purpose:

To predict, prevent and potentially treat glaucoma

2. Benefit:

Treatments are limited and there is no cure for glaucoma. In this study, there will be the potential to examine a wide range of questions, including how genes and the environment influence one’s eye health; specifically, how these factors impact the determinants of glaucoma/pseudoexfoliation glaucoma in individuals that live in Guatemala. Understanding how genes and the environment influence glaucoma and how these factors interact to cause glaucoma and the leading causes of blindness globally, should hopefully lead to effective preventive and therapeutic targets.

Participation in this study is entirely voluntary. Your decision not to participate will not have any negative effect on you or your medical care.

3. Participation

If you agree to participate in this research study, we will ask you to donate a blood sample. Your “master stored sample” will contain your first and last name along with the unique sample identification number. This master stored sample will be stored in −80 °C freezer under lock and key and a 24-alarm system. The computer database that contains this information is user- and password-protected and maintained to organize the large number of samples entered into the DeAngelis laboratory, sorted according to your diagnosis. All working stocks, bench work analysis, and subsequent results are de-identified, meaning that your name is not associated with the unique identifying number. This information will be retained in a secured database customized for rapidly recording results. The database will be shared among researchers in the DeAngelis laboratory and is accessible through any PC in the laboratory. It is password-protected so that each investigator can view results entered by other lab members but can add or edit results only for the genes/variants/studies on which the user is working.

4. POTENTIAL BENEFITS

There are no direct benefits to you for participating in this research study. However, if genes and/or other factors that have a role in the diseases of study are discovered, they may help scientists find therapies to treat these diseases. It should be emphasized that even if this research is successful, it will take many years for it to lead to a new therapy.

Blood Draw: All individuals: EDTA 2 (10 mL purple top) tubes

10 mL Purple Top

10 mL Purple Top

5. Labeling of tubes:

Patient’s first name, middle initial and last name (this will later be de-identified and assigned a unique number).

Patient’s date of birth and sex.

Date of Collection.
